# Supplementary material for: Immune escape and attenuated severity associated with the SARS-CoV-2 BA.2.86/JN.1 lineage
Source: Nat Commun. 2024 Oct 3;15:8550. doi: 10.1038/s41467-024-52668-w (PMC11450198; doi:10.1038/s41467-024-52668-w)
Supplement: Supplementary file 1 — Supplementary Information [file 41467_2024_52668_MOESM1_ESM.pdf]

SUPPORTING INFORMATION

Immune escape and attenuated severity associated with the SARS-CoV-2 BA.2.86/JN.1 lineage

Authors: Joseph A. Lewnard[1,*], Parag Mahale[2], Debbie Malden[2], Vennis Hong[2], Bradley K. Ackerson[2], Bruno J. Lewin[2], Ruth Link-Gelles[3], Leora R. Feldstein[4], Marc Lipsitch[4], Sara Y. Tartof[2,5]

Affiliations:
1. School of Public Health, University of California, Berkeley, Berkeley, California, United States
2. Department of Research & Evaluation, Kaiser Permanente Southern California, Pasadena, California, United States
3. Coronavirus and Other Respiratory Viruses Division, National Center for Immunization and Respiratory Diseases, US Centers for Disease Control & Prevention, Atlanta, Georgia, United States
4. COVID-19 Response Team, Centers for Disease Control and Prevention, Atlanta, Georgia, United States
5. Department of Health Systems Science, Kaiser Permanente Bernard J. Tyson School of Medicine, Pasadena, California, United States

Contents of this supplement

Item	Title	Page
Table S1	Characteristics of outpatient cases tested on ThermoFisher TaqPath COVID-19 Combo Kit (TF) and other assays.	2
Table S2	S-gene target detection and infecting lineage among outpatient-diagnosed cases with samples processed using the TaqPath COVID-19 Combo Kit assay.	3
Table S3	Prior vaccination and documented SARS-CoV-2 infection among cases infected with JN.1 and non-JN.1 lineages.	4
Table S4	Putative prior infecting variants among individuals infected with SARS-CoV-2 according to lineage.	5
Table S5	Comparison of estimates for lineage-based and period-based comparisons.	6
Table S6	Diagnosis codes used to identify acute respiratory infection-associated emergency department presentations and hospital admissions.	7
Table S7	Clinical progression among cases according to infecting lineage, restricting the sample to cases with >5 healthcare interactions in the year preceding their index test.	9
Table S8	Association of vaccination with prior documented infection.	10
Table S9	Comparison of complete case analysis and single imputation results for clinical progression among individuals infected with SARS-CoV-2 according to infecting lineage.	11
Table S10	Comparison of complete case analysis and single imputation results for prior vaccination and documented SARS-CoV-2 infection among individuals infected with SARS-CoV-2 according to infecting lineage.	12
Figure S1	Sensitivity analyses addressing the independent association of BA.2.86 lineage detection with prior vaccination, accounting for additional unobserved infections overall, among cases infected with BA.2.86 lineages, and who did not experience progression (emergency department presentation).	13
Figure S2	Sensitivity analyses addressing the independent association of BA.2.86 lineage detection with prior vaccination, accounting for additional unobserved infections overall, among cases infected with BA.2.86 lineages, and who did not experience progression (hospital admission).	14
Figure S3	Sensitivity analyses addressing the independent association of BA.2.86 lineage detection with prior vaccination, accounting for additional unobserved infections according to vaccination status.	15
Figure S4	Kaplan-Meier plots for clinical progression analyses comparing cases infected with BA.2.86 lineages to non-BA.2.86 lineages.	16
Figure S5	Observed and fitted proportions of cases infected with BA.2.86 lineages.	17

Table S1: Characteristics of outpatient cases tested on ThermoFisher TaqPath COVID-19 Combo Kit (TF) and other assays.

Characteristic		Cases, *n*/*N* (%)	
		Primary analytic cohort (tested on TF assays)	Cases tested on non-TF assays
		N=7,694	*N*=38,373
Age (years)[1]			
	0-9	323 (4.2)	1,056 (2.8)
	10-19	387 (5.0)	1,406 (3.7)
	20-29	737 (9.6)	2,995 (7.8)
	30-39	1,273 (16.5)	5,679 (14.8)
	40-49	1,424 (18.5)	6,502 (16.9)
	50-59	1,359 (17.7)	6,726 (17.5)
	60-69	1,117 (14.5)	6,408 (16.7)
	70-79	698 (9.1)	4,883 (12.7)
	≥80	376 (4.9)	2,718 (7.1)
Sex			
	Female	4,648 (60.4)	23,711 (61.8)
	Male	3,046 (39.6)	14,662 (38.2)
Race			
	White, non-Hispanic	1,669 (21.7)	13,180 (34.3)
	Black, non-Hispanic	803 (10.4)	2,607 (6.8)
	Hispanic (any race)	3,661 (47.6)	15,371 (40.1)
	Asian	1,014 (13.2)	4,976 (12.5)
	Pacific Islander	75 (1.0)	294 (0.8)
	Other/mixed/unknown race	472 (5.5)	2,125 (6.1)
Body mass index[1]			
	Underweight (<18.5)	280 (3.6)	1,204 (3.1)
	Normal weight (18.5-24.9)	1,583 (20.6)	7,838 (20.4)
	Overweight (25.0-29.9)	2,077 (27.0)	10,329 (26.9)
	Obese (≥30.0)	2,907 (37.8)	14,533 (37.9)
Cigarette smoking[1]			
	Never smoker	5,395 (70.1)	25,622 (66.8)
	Former smoker	1,366 (17.8)	7,530 (19.6)
	Current smoker	283 (3.7)	1,354 (3.5)
Charlson comorbidity index			
	0	4,740 (61.6)	21,568 (56.2)
	1-2	2,098 (27.3)	11,218 (29.2)
	3-5	616 (8.0)	2,819 (10.0)
	≥6	240 (3.1)	1,768 (4.6)
Prior-year healthcare utilization			
	0-9 outpatient encounters	3,979 (51.7)	19,234 (50.1)
	10-19 outpatient encounters	1,991 (25.9)	10,352 (27.0)
	20-29 outpatient encounters	893 (11.6)	4,412 (11.5)
	≥30 outpatient encounters	831 (10.8)	4,375 (11.4)
	Any emergency department presentation	1,682 (21.9)	9,002 (23.5)
	Any inpatient admission	418 (5.4)	2,573 (6.7)
Census tract household median income[1]			
	<$40,000	347 (4.5)	1,421 (3.7)
	$40,000-79,999	3,054 (39.7)	13,379 (34.9)
	$80,000-119,999	2,579 (33.5)	13,555 (35.3)
	$120,000-159,999	1,080 (14.0)	6,345 (16.5)
	≥$160,000	361 (4.7)	2,242 (5.8)
Receipt of nirmatrelvir-ritonavir			
	Received within ≤5 days from diagnosis	1,455 (18.9)	6,239 (16.3)
	Received >5 days from diagnosis	25 (0.3)	212 (0.6)
	Not received	6,214 (80.8)	31,922 (83.2)

[1]Counts and percentages are counted excluding missing values.

Table S2: S-gene target detection and infecting lineage among outpatient-diagnosed cases with samples processed using the TaqPath COVID-19 Combo Kit assay.

Lineage detected by sequencing		S-gene detection outcome by TaqPath assay, *n* (%)	
		S-gene target failure	S-gene target detected
		N=582	*N*=496
JN.1. and related BA.2.86 or BA.2 recombinant lineages			
	JN.1	455 (78.2)	18 (3.6)
	Unspecified BA.2.86 recombinant	113 (19.4)	2 (0.4)
		Sensitivity excluding unspecified BA.2. recombinant lineages: 97.6% (568/582)	
		Specificity excluding unspecified BA.2 recombinant lineages: 96.0% (476/496)	
	Unspecified BA.2 recombinant	14 (2.4)	2 (0.4)
		Sensitivity excluding unspecified BA.2. recombinant lineages: 100% (582/582)	
		Specificity excluding unspecified BA.2 recombinant lineages: 96.3% (478/496)	
Other lineages			
	EG.5 recombinant	0	77 (15.5)
	HK.3 recombinant	0	42 (8.5)
	HV.1 recombinant	0	168 (33.9)
	JD.1 recombinant	0	47 (9.5)
	JG.3 recombinant	0	48 (9.7)
	Unspecified XBB recombinant	0	90 (18.1)
	Other	0	2 (0.4)

Table S3: Prior vaccination and documented SARS-CoV-2 infection among cases infected with JN.1 and non-JN.1 lineages.

Infection history	Exposure	Cases, *n*/*N* (%)		Odds ratio (95% CI), infection with BA.2.86-derived lineage versus non-BA.2.86 lineage	
		S-gene detected (non-BA.2.86 lineage)	S-gene target failure (BA.2.86-derived lineage)	Unadjusted[1]	Adjusted[2]
≥1 documented infection		*N*=2,109	*N*=1,574		
	0 vaccine doses	244 (11.6)	141 (9.0)	ref.	ref.
	1 vaccine dose	57 (2.7)	36 (2.3)	1.05 (0.73, 1.52)	1.03 (0.71, 1.49)
	2 vaccine doses	464 (22.0)	301 (19.1)	1.05 (0.86, 1.28)	1.01 (0.82, 1.24)
	3 vaccine doses	754 (35.8)	575 (36.5)	1.18 (0.98, 1.420	1.15 (0.95, 1.40)
	4 vaccine doses	348 (16.5)	307 (19.5)	1.25 (1.02, 1.53)	1.24 (0.94, 1.63)
	≥5 vaccine doses	242 (11.5)	214 (13.6)	1.24 (1.00, 1.53)	1.38 (1.00, 1.89)
≥2 documented infections		*N*=356	*N*=305		
	0 vaccine doses	47 (13.2)	31 (10.2)	ref.	ref.
	1 vaccine dose	7 (2.0)	9 (3.0)	– –	– –
	2 vaccine doses	85 (23.9)	57 (18.7)	1.00 (0.65, 1.56)	0.96 (0.61, 1.51)
	3 vaccine doses	135 (37.9)	120 (39.3)	1.19 (0.80, 1.78)	1.13 (0.75, 1.70)
	4 vaccine doses	58 (16.3)	61 (20.0)	1.26 (0.81, 1.94)	1.37 (0.78, 2.41)
	≥5 vaccine doses	24 (6.7)	27 (8.9)	1.27 (0.76, 2.14)	1.66 (0.81, 3.41)

Data encompass the primary analytic cohort, comprised of individuals testing positive for SARS-CoV-2 from tests undertaken in outpatient settings between 1 December, 2023 and 30 January, 2024 which were processed via TaqPath COVID-19 Combo Kit assays, who belonged to KPSC health plans for at least one year prior to their index test date.
[1]Unadjusted odds ratios are computed via conditional logistic regression models matching on week of testing alone.
[2]Adjusted odds ratios are computed via conditional logistic regression models matching on week of testing and controlling for age, sex, race/ethnicity, body mass index, history of cigarette smoking, prior-year healthcare utilization across all settings, Charlson comorbidity index, and median household income within cases' census tract according to the categorization scheme indicated in **Table 1**. Missing values were addressed via multiple imputation, with results pooled across 5 pseudo-dataset replicates.

Table S4: Putative prior infecting variants among individuals infected with SARS-CoV-2 according to lineage.

Infection history	Cases, *n*/*N* (%)		Odds ratio (95% CI), infection with BA.2.86-derived lineage versus non-BA.2.86 lineage	
	S-gene detected (non-BA.2.86 lineage)	S-gene target failure (BA.2.86-derived lineage)	Unadjusted[1]	Adjusted
	N=4,614	*N*=3,080		
0 documented infections	2,505 (54.3)	1,506 (48.9)	ref.	ref.
Prior documented infection during pre-Delta period[1]	572 (12.4)	378 (12.3)	1.00 (0.90, 1.12)	1.00 (0.89, 1.12)
Prior documented infection during Delta period[2]	155 (3.4)	102 (3.3)	0.97 (0.80, 1.19)	0.95 (0.77, 1.16)
Prior documented infection during BA.1 period[3]	621 (13.5)	465 (15.1)	1.08 (0.98, 1.20)	1.08 (0.97, 1.19)
Prior documented infection during BA.2 period[4]	303 (6.6)	267 (8.7)	1.17 (1.03, 1.33)	1.16 (1.02, 1.32)
Prior documented infection during BA.4/BA.5 period[5]	559 (12.1)	414 (13.4)	1.07 (0.96, 1.18)	0.98 (0.87, 1.11)
Prior documented infection during XBB period[6]	269 (5.8)	264 (8.6)	1.18 (1.04, 1.34)	1.16 (1.02, 1.32)

Data encompass the primary analytic cohort, comprised of individuals testing positive for SARS-CoV-2 from tests undertaken in outpatient settings between 1 December, 2023 and 30 January, 2024 which were processed via TaqPath COVID-19 Combo Kit assays, who belonged to KPSC health plans for at least one year prior to their index test date.

[1]Unadjusted odds ratios are computed via conditional logistic regression models matching on week of testing alone.

[2]Adjusted odds ratios are computed via conditional logistic regression models matching on week of testing and controlling for age, sex, race/ethnicity, body mass index, history of cigarette smoking, prior-year healthcare utilization across all settings, Charlson comorbidity index, and median household income within cases' census tract according to the categorization scheme indicated in **Table 1**. Missing values were addressed via multiple imputation, with results pooled across 5 pseudo-dataset replicates.

[3]Pre-Delta period: 1 January, 2020 to 19 June, 2021.

[4]Delta period: 20 June, 2021 to 19 December, 2021.

[5]BA.1 period: 20 December, 2021 to 2 February, 2022.

[6]BA.2 period: 3 February, 2022 to 24 June, 2022.

[7]BA.4/BA.5 period: 25 June, 2022 to 30 November, 2022.

[8]XBB period: 1 December, 2022 to 31 October, 2023.

Table S5: Comparison of estimates for lineage-based and period-based comparisons.

Characteristic or outcome and measure of association	Lineage-based analysis (for BA.2.86 vs. non-BA.2.86 infection)[1]	Period-based analysis (for specified period vs. Nov. 1-30, 2023)[2]			
		Dec. 1-15, 2023	Dec. 16-31, 2023	Jan. 1-15, 2024	Jan. 16-31, 2024
Number of prior COVID-19 vaccine doses, aOR (95% confidence interval)[3]					
0 doses	ref.	ref.	ref.	ref.	ref.
5 doses	1.43 (1.20, 1.71)	0.95 (0.87, 1.04)	1.01 (0.93, 1.09)	1.04 (0.95, 1.13)	0.99 (0.89, 1.10)
6 doses	1.57 (1.28, 1.91)	1.03 (0.91, 1.17)	1.10 (1.00, 1.22)	1.12 (1.0,0, 1.26)	1.16 (1.01, 1.33)
≥7 doses	1.69 (1.16, 2.45)	1.10 (0.84, 1.43)	1.24 (1.03, 1.50)	1.30 (1.04, 1.63)	1.45 (1.13, 1.87)
Prior documented SARS-CoV-2 infections, aOR (95% confidence interval)[3]					
0 prior infections	ref.	ref.	ref.	ref.	ref.
1 prior infection	1.08 (1.00, 1.17)	1.07 (1.03, 1.12)	1.09 (1.05, 1.13)	1.10 (1.06, 1.14)	1.13 (1.07, 1.18)
2 prior infections	1.14 (0.99, 1.30)	1.03 (0.95, 1.12)	1.06 (0.98, 1.13)	1.12 (1.03, 1.21)	1.17 (1.07, 1.29)
≥3 prior infections	1.30 (0.89, 1.91)	0.96 (0.73, 1.26)	1.13 (0.93, 1.37)	1.25 (1.00, 1.55)	1.28 (0.98, 1.67)
Progression to illness requiring higher-level care delivery, aHR (95% confidence interval)[4]					
Emergency department presentation due to any cause	0.47 (0.31, 0.70)	1.13 (0.99, 1.29)	1.13 (1.00, 1.28)	1.05 (0.91, 1.21)	0.57 (0.39, 0.82)
Emergency department presentation with ARI diagnosis	0.40 (0.14, 1.13)	0.91 (0.67, 1.23)	0.96 (0.73, 1.26)	0.68 (0.47, 0.97)	0.32 (0.10, 1.04)
Hospital admission due to any cause	0.50 (0.22, 1.13)	0.97 (0.79, 1.19)	0.91 (0.75, 1.10)	0.59 (0.43, 0.80)	0.70 (0.33, 1.46)
Hospital admission with ARI diagnosis	0.13 (0.02, 1.04)	0.85 (0.62, 1.16)	0.85 (0.63, 1.14)	0.26 (0.14, 0.49)	0.16 (0.05, 1.17)

[1]Data encompass the primary analytic cohort, comprised of individuals testing positive for SARS-CoV-2 from tests undertaken in outpatient settings between 1 December, 2023 and 30 January, 2024 which were processed via TaqPath COVID-19 Combo Kit assays, who belonged to KPSC health plans for at least one year prior to their index test date.

[2]Data encompass the individuals testing positive for SARS-CoV-2 from tests undertaken in outpatient settings between 1 November, 2023 and 30 January, 2024 in any outpatient setting (without restriction on the assay used for test processing), for patients who belonged to KPSC health plans for at least one year prior to their index test date.

[3]Adjusted odds ratios are computed via logistic regression models controlling for age, sex, race/ethnicity, body mass index, history of cigarette smoking, prior-year healthcare utilization across all settings, Charlson comorbidity index, and median household income within cases' census tract according to the categorization scheme indicated in **Table 1**. Missing values were addressed via multiple imputation, with results pooled across 5 pseudo-dataset replicates. For comparisons of cases infected with BA.2.86 lineages vs. non-BA.2.86 lineages, cases were matched on week of testing via conditional logistic regression models.

[4]Adjusted hazard ratios are computed via Cox proportional hazards regression models controlling for age, sex, race/ethnicity, body mass index, history of cigarette smoking, prior-year healthcare utilization across all settings, Charlson comorbidity index, and median household income within cases' census tract according to the categorization scheme indicated in **Table 1**. In addition, nirmatrelvir-ritonavir receipt is defined as a time-varying exposure. Missing values were addressed via multiple imputation, with results pooled across 5 pseudo-dataset replicates. For comparisons of cases infected with BA.2.86 lineages vs. non-BA.2.86 lineages, cases were matched on week of testing.

Table S6: Diagnosis codes used to identify acute respiratory infection-associated emergency department presentations and hospital admissions.

ICD-10-CM Code	Diagnosis
A48.1	Legionnaire's disease
B34.2	Coronavirus infection (unspecified)
B44.0	Invasive pulmonary aspergillosis
B97.29	Other coronavirus as the cause of diseases classified elsewhere
J00	Acute nasopharyngitis (common cold)
J01.00	Acute maxillary sinusitis, unspecified
J01.10	Acute frontal sinusitis, unspecified
J01.20	Acute ethmoidal sinusitis, unspecified
J01.30	Acute sphenoidal sinusitis, unspecified
J01.40	Acute pansinusitis, unspecified
J01.80	Other acute sinusitis
J01.90	Acute sinusitis, unspecified
J02.0	Streptococcal pharyngitis
J02.8	Acute pharyngitis due to other specified organisms
J02.9	Acute pharyngitis, unspecified
J03.00	Acute streptococcal tonsillitis, unspecified
J03.90	Acute tonsillitis, unspecified
J04.0	Acute laryngitis
J04.10	Acute tracheitis without obstruction
J05.0	Acute obstructive laryngitis (croup)
J05.10	Acute epiglottitis without obstruction
J06.0	Acute laryngopharyngitis
J06.9	Acute upper respiratory infection, unspecified
J09.X1	Influenza due to identified novel influenza A virus with pneumonia
J09.X2	Influenza due to identified novel influenza A virus with other respiratory manifestations
J10.00	Influenza due to other identified influenza virus with unspecified type of pneumonia
J10.01	Influenza due to other identified influenza virus with same other identified influenza virus pneumonia
J10.08	Influenza due to other identified influenza virus with other pneumonia
J10.1	Influenza due to other identified influenza virus with other respiratory manifestations
J10.2	Influenza due to other identified influenza virus with gastrointestinal manifestations
J11.00	Influenza due to unidentified influenza virus with unspecified type of pneumonia
J11.08	Influenza due to unidentified influenza virus with specified pneumonia
J11.1	Influenza due to unidentified influenza virus with other respiratory manifestations
J12.1	Respiratory syncytial virus pneumonia
J12.2	Parainfluenza virus pneumonia
J12.3	Human metapneumovirus pneumonia
J12.81	Pneumonia due to SARS-associated coronavirus
J12.82	Pneumonia due to coronavirus disease 2019
J12.89	Other viral pneumonia
J12.9	Viral pneumonia, unspecified
J13	Pneumonia due to *Streptococcus pneumoniae*
J14	Pneumonia due to *Haemophilus influenzae*
J15.0	Pneumonia due to *Klebsiella pneumoniae*
J15.1	Pneumonia due to *Pseudomonas*
J15.20	Pneumonia due to *Staphylococcus*, unspecified
J15.211	Pneumonia due to methicillin susceptible *Staphylococcus aureus*
J15.212	Pneumonia due to methicillin resistant *Staphylococcus aureus*
J15.4	Pneumonia due to other *Streptococci*
J15.5	Pneumonia due to *Escherichia coli*
J15.6	Pneumonia due to other aerobic gram-negative bacteria
J15.7	Pneumonia due to *Mycoplasma pneumoniae*
J15.8	Pneumonia due to other specified bacteria
J15.9	Unspecified bacterial pneumonia
J16.8	Pneumonia due to other specified infectious organisms
J18.0	Bronchopneumonia, unspecified organism
J18.1	Lobar pneumonia, unspecified organism
J18.8	Other pneumonia, unspecified organism
J18.9	Pneumonia, unspecified organism
J20.2	Acute bronchitis due to *Streptococcus*
J20.5	Acute bronchitis due to respiratory syncytial virus
J20.6	Acute bronchitis due to rhinovirus
J20.8	Acute bronchitis due to other specified organisms
J20.9	Acute bronchitis, unspecified
J22	Unspecified acute lower respiratory infection
J39.0	Retropharyngeal and parapharyngeal abscess
J39.1	Other abscess of pharynx
J39.2	Other diseases of pharynx
J39.8	Other specified diseases of upper respiratory tract
J80	Acute respiratory distress syndrome
J96.00	Acute respiratory failure, unspecified with hypoxia or hypercapnia
J96.01	Acute respiratory failure with hypoxia

J96.02	Acute respiratory failure with hypercapnia
J96.10	Chronic respiratory failure, unspecified with hypoxia or hypercapnia
J96.11	Chronic respiratory failure with hypoxia
J96.12	Chronic respiratory failure with hypercapnia
J96.20	Acute and chronic respiratory failure, unspecified with hypoxia or hypercapnia
J96.21	Acute and chronic respiratory failure with hypoxia
J96.22	Acute and chronic respiratory failure with hypercapnia
J96.90	Respiratory failure, unspecified with hypoxia or hypercapnia
J96.91	Respiratory failure with hypoxia
J96.92	Respiratory failure with hypercapnia
M35.81	Multisystem inflammatory syndrome
M35.89	Other specified systemic involvement of connective tissue
R05.1	Acute cough
R05.3	Chronic cough
R05.8	Other specified cough
R05.9	Cough, unspecified
R09.2	Respiratory arrest
R50.9	Fever, unspecified
U07.1	COVID-19

Table S7: Clinical progression among cases according to infecting lineage, restricting the sample to cases with >5 healthcare interactions in the year preceding their index test.

**Episode type**	**Outcome**	**Events, *n* (Rate per 10,000 days)**		**Hazard ratio (95% CI), JN.1 vs. non-JN.1 infection**	
		S-gene detected (non-BA.2.86 lineage)	S-gene target failure (BA.2.86-derived lineage)	Unadjusted[1]	Adjusted[2]
		N=3,147	*N*=2,020		
Episodes associated with all causes					
	Emergency department presentation	104 (30.0)	29 (15.3)	0.46 (0.30, 0.71)	0.50 (0.33, 0.77)
	Hospital admission	33 (5.5)	5 (1.7)	0.27 (0.10, 0.70)	0.35 (0.13, 0.96)
	ICU admission, mechanical ventilation, or death	9 (1.2)	1 (0.3)	0.21 (0.03, 1.71)	– –
	Death	5 (0.7)	1 (0.3)	0.39 (0.04, 3.41)	– –
ARI-associated episodes[3]					
	Emergency department presentation	21 (6.0)	5 (2.6)	0.37 (0.14, 1.00)	0.39 (0.14, 1.11)
	Hospital admission	16 (2.7)	1 (0.3)	0.10 (0.01, 0.79)	0.12 (0.01, 0.93)

Data encompass the primary analytic cohort, comprised of individuals testing positive for SARS-CoV-2 from tests undertaken in outpatient settings between 1 December, 2023 and 30 January, 2024 which were processed via TaqPath COVID-19 Combo Kit assays, who belonged to KPSC health plans for at least one year prior to their index test date.

[1]Unadjusted hazard ratios are computed via Cox proportional hazards regression models matching on week of testing alone.

[2]Adjusted hazard ratios are computed via Cox proportional hazards regression models matching on week of testing and controlling for age, sex, race/ethnicity, body mass index, history of cigarette smoking, prior-year healthcare utilization across all settings, Charlson comorbidity index, and median household income within cases' census tract according to the categorization scheme indicated in **Table 1**. In addition, nirmatrelvir-ritonavir receipt is defined as a time-varying exposure. Missing values were addressed via multiple imputation, with results pooled across 5 pseudo-dataset replicates.

[3]Acute respiratory infection diagnosis codes are presented in **Table S6**.

Table S8: Association of vaccination with prior documented infection.

Exposure		Effect measure	
		Adjusted odds ratio, any prior documented infection (95% CI)[1]	Adjusted risk ratio, number of prior documented infections (95% CI)[2]
Receipt of updated COVID-19 vaccines[3]			
	No XBB.1.5 (monovalent) vaccine doses	ref.	ref.
	Any XBB.1.5 (monovalent) vaccine doses	0.85 (0.75, 0.96)	0.37 (0.29, 0.48)
	No BA.4/BA.5 (bivalent) vaccine doses	ref.	ref.
	Any BA.4/BA.5 (bivalent) vaccine doses	0.89 (0.82, 0.97)	0.83 (0.74, 0.93)
	0 Omicron-targeted vaccine doses	ref.	ref.
	Any Omicron-targeted vaccine	0.86 (0.79, 0.93)	0.37 (0.29, 0.48)
	Both BA.4/BA.5 (bivalent) and XBB1.5 (monovalent) vaccines	0.76 (0.66, 0.86)	0.37 (0.29, 0.48)
Number of vaccine doses received			
	0 vaccine doses	ref.	ref.
	5 vaccine doses	0.65 (0.55, 0.77)	0.80 (0.71, 0.91)
	6 vaccine doses	0.67 (0.55, 0.82)	0.59 (0.50, 0.69)
	≥7 vaccine doses	0.94 (0.66, 1.36)	0.61 (0.51, 0.73)

Data encompass the primary analytic cohort, comprised of individuals testing positive for SARS-CoV-2 from tests undertaken in outpatient settings between 1 December, 2023 and 30 January, 2024 which were processed via TaqPath COVID-19 Combo Kit assays, who belonged to KPSC health plans for at least one year prior to their index test date.

[1]Adjusted odds ratios are computed via conditional logistic regression models matching on week of testing and controlling for detection of BA.2.86 lineages, age, sex, race/ethnicity, body mass index, history of cigarette smoking, prior-year healthcare utilization across all settings, Charlson comorbidity index, and median household income within cases' census tract according to the categorization scheme indicated in **Table 1**. Missing values were addressed via multiple imputation, with results pooled across 5 pseudo-dataset replicates.

[2]Adjusted risk ratios are computed via Poisson regression models controlling for week of testing, detection of BA.2.86 lineages, age, sex, race/ethnicity, body mass index, history of cigarette smoking, prior-year healthcare utilization across all settings, Charlson comorbidity index, and median household income within cases' census tract according to the categorization scheme indicated in **Table 1**. Missing values were addressed via multiple imputation, with results pooled across 5 pseudo-dataset replicates.

[3]Analyses of vaccine type and timing adjust for number of monovalent wild-type (Wuhan-Hu-1) vaccine doses received.

Table S9: Comparison of complete case analysis and single imputation results for clinical progression among individuals infected with SARS-CoV-2 according to infecting lineage.

Episode type	Outcome	Adjusted hazard ratio (95% CI), JN.1 vs. non-JN.1 infection[1]					
		Complete case analysis	Imputation 1	Imputation 2	Imputation 3	Imputation 4	Imputation 5
Episodes associated with all causes							
	Emergency department presentation	0.47 (0.31, 0.71)	0.47 (0.31, 0.70)	0.47 (0.31, 0.70)	0.47 (0.31, 0.70)	0.47 (0.31, 0.69)	0.47 (0.31, 0.69)
	Hospital admission	0.45 (0.18, 1.11)	0.50 (0.22, 1.14)	0.49 (0.22, 1.14)	0.50 (0.22, 1.14)	0.49 (0.22, 1.13)	0.49 (0.22, 1.12)
ARI-associated episodes[2]							
	Emergency department presentation	0.39 (0.14, 1.12)	0.40 (0.14, 1.14)	0.40 (0.14, 1.14)	0.40 (0.14, 1.12)	0.40 (0.14, 1.12)	0.40 (0.14, 1.11)
	Hospital admission	0.13 (0.02, 1.03)	0.13 (0.02, 1.06)	0.13 (0.02, 1.05)	0.13 (0.02, 1.02)	0.14 (0.02, 1.11)	0.12 (0.02, 0.96)

Data encompass the primary analytic cohort, comprised of individuals testing positive for SARS-CoV-2 from tests undertaken in outpatient settings between 1 December, 2023 and 30 January, 2024 which were processed via TaqPath COVID-19 Combo Kit assays, who belonged to KPSC health plans for at least one year prior to their index test date.

[1]Adjusted hazard ratios are computed via Cox proportional hazards regression models matching on week of testing and controlling for age, sex, race/ethnicity, body mass index, history of cigarette smoking, prior-year healthcare utilization across all settings, Charlson comorbidity index, and median household income within cases' census tract according to the categorization scheme indicated in **Table 1**. In addition, nirmatrelvir-ritonavir receipt is defined as a time-varying exposure.

[2]Acute respiratory infection diagnosis codes are presented in **Table S6**.

Table S10: Comparison of complete case analysis and single imputation results for prior vaccination and documented SARS-CoV-2 infection among individuals infected with SARS-CoV-2 according to infecting lineage.

Exposure		Adjusted odds ratio (95% CI), JN.1 vs. non-JN.1 infection[1]					
		Complete case analysis	Imputation 1	Imputation 2	Imputation 3	Imputation 4	Imputation 5
Prior vaccination							
	0 vaccine doses	ref.	ref.	ref.	ref.	ref.	ref.
	1 vaccine dose	0.89 (0.64, 1.25)	1.05 (0.80, 1.38)	1.05 (0.80, 1.37)	1.05 (0.80, 1.38)	1.04 (0.79, 1.37)	1.05 (0.80, 1.38)
	2 vaccine doses	1.08 (0.91, 1.29)	1.10 (0.94, 1.28)	1.10 (0.94, 1.28)	1.10 (0.94, 1.28)	1.10 (0.94, 1.28)	1.10 (0.94, 1.28)
	3 vaccine doses	1.18 (1.01, 1.39)	1.20 (1.04, 1.39)	1.20 (1.04, 1.39)	1.20 (1.04, 1.39)	1.20 (1.04, 1.39)	1.21 (1.04, 1.39)
	4 vaccine doses	1.19 (1.00, 1.41)	1.24 (1.06, 1.45)	1.23 (1.06, 1.44)	1.23 (1.05, 1.44)	1.23 (1.05, 1.44)	1.24 (1.06, 1.45)
	5 vaccine doses	1.40 (1.15, 1.71)	1.43 (1.20, 1.71)	1.43 (1.20, 1.70)	1.42 (1.19, 1.70)	1.43 (1.19, 1.71)	1.43 (1.20, 1.71)
	6 vaccine doses	1.57 (1.27, 1.95)	1.57 (1.28, 1.91)	1.57 (1.28, 1.91)	1.56 (1.28, 1.91)	1.56 (1.28, 1.91)	1.57 (1.29, 1.92)
	≥7 vaccine doses	1.65 (1.12, 2.44)	1.69 (1.16, 2.45)	1.69 (1.16, 2.45)	1.70 (1.17, 2.45)	1.69 (1.16, 2.44)	1.69 (1.17, 2.45)
Receipt of Omicron-targeted vaccines[2]							
	0 Omicron-targeted vaccine doses	ref.	ref.	ref.	ref.	ref.	ref.
	Any Omicron-targeted vaccine	1.10 (1.01, 1.21)	1.12 (1.03, 1.22)	1.12 (1.02, 1.21)	1.11 (1.02, 1.21)	1.12 (1.03, 1.22)	1.12 (1.03, 1.21)
	Both BA.4/BA.5 (bivalent) and XBB1.5 (monovalent) vaccines	1.30 (1.14, 1.49)	1.28 (1.13, 1.46)	1.28 (1.13, 1.45)	1.28 (1.13, 1.45)	1.28 (1.13, 1.46)	1.28 (1.13, 1.45)
	No BA.4/BA.5 (bivalent) vaccine doses	ref.	ref.	ref.	ref.	ref.	ref.
	Any BA.4/BA.5 (bivalent) vaccine doses	1.08 (0.98, 1.19)	1.10 (1.01, 1.20)	1.10 (1.01, 1.20)	1.10 (1.00, 1.20)	1.10 (1.01, 1.20)	1.10 (1.01, 1.20)
	No XBB.1.5 (monovalent) vaccine doses	ref.	ref.	ref.	ref.	ref.	ref.
	Any XBB.1.5 (monovalent) vaccine doses	1.17 (1.03, 1.33)	1.14 (1.01, 1.28)	1.14 (1.01, 1.28)	1.14 (1.01, 1.28)	1.14 (1.01, 1.28)	1.14 (1.01, 1.28)
Timing of prior vaccination[2]							
	No doses received	ref.	ref.	ref.	ref.	ref.	ref.
	Last vaccine dose within <3 months	1.04 (0.77, 1.41)	1.05 (0.80, 1.38)	1.05 (0.80, 1.38)	1.05 (0.80, 1.38)	1.05 (0.80, 1.39)	1.05 (0.80, 1.38)
	Last vaccine dose within 3-6 months	1.12 (0.78, 1.59)	1.10 (0.79, 1.52)	1.09 (0.79, 1.52)	1.09 (0.79, 1.51)	1.09 (0.79, 1.52)	1.09 (0.79, 1.51)
	Last vaccine dose >6 months prior	0.95 (0.77, 1.17)	0.97 (0.81, 1.17)	0.97 (0.81, 1.17)	0.97 (0.81, 1.17)	0.97 (0.81, 1.17)	0.97 (0.81, 1.17)
Documented prior infection							
	0 documented infections	ref.	ref.	ref.	ref.	ref.	ref.
	Any prior infection	1.09 (1.01, 1.19)	1.09 (1.02, 1.18)	1.09 (1.02, 1.18)	1.09 (1.01, 1.18)	1.09 (1.01, 1.18)	1.09 (1.02, 1.18)
	1 documented infection	1.07 (0.98, 1.17)	1.08 (1.00, 1.17)	1.08 (1.00, 1.17)	1.08 (1.00, 1.17)	1.08 (1.00, 1.17)	1.08 (1.00, 1.17)
	2 documented infections	1.18 (1.02, 1.36)	1.14 (0.99, 1.29)	1.14 (1.00, 1.30)	1.13 (0.99, 1.29)	1.13 (0.99, 1.29)	1.14 (1.00, 1.30)
	≥3 documented infections	1.45 (0.99, 2.14)	1.30 (0.88, 1.91)	1.31 (0.90, 1.94)	1.30 (0.88, 1.90)	1.30 (0.89, 1.91)	1.30 (0.89, 1.92)

Data encompass the primary analytic cohort, comprised of individuals testing positive for SARS-CoV-2 from tests undertaken in outpatient settings between 1 December, 2023 and 30 January, 2024 which were processed via TaqPath COVID-19 Combo Kit assays, who belonged to KPSC health plans for at least one year prior to their index test date.

[1]Adjusted odds ratios are computed via conditional logistic regression models matching on week of testing and controlling for age, sex, race/ethnicity, body mass index, history of cigarette smoking, prior-year healthcare utilization across all settings, Charlson comorbidity index, and median household income within cases' census tract according to the categorization scheme indicated in **Table 1**. Missing values were addressed via multiple imputation, with results pooled across 5 pseudo-dataset replicates.

[2]Analyses of vaccine type and timing adjust for number of monovalent wild-type (Wuhan-Hu-1) vaccine doses received.

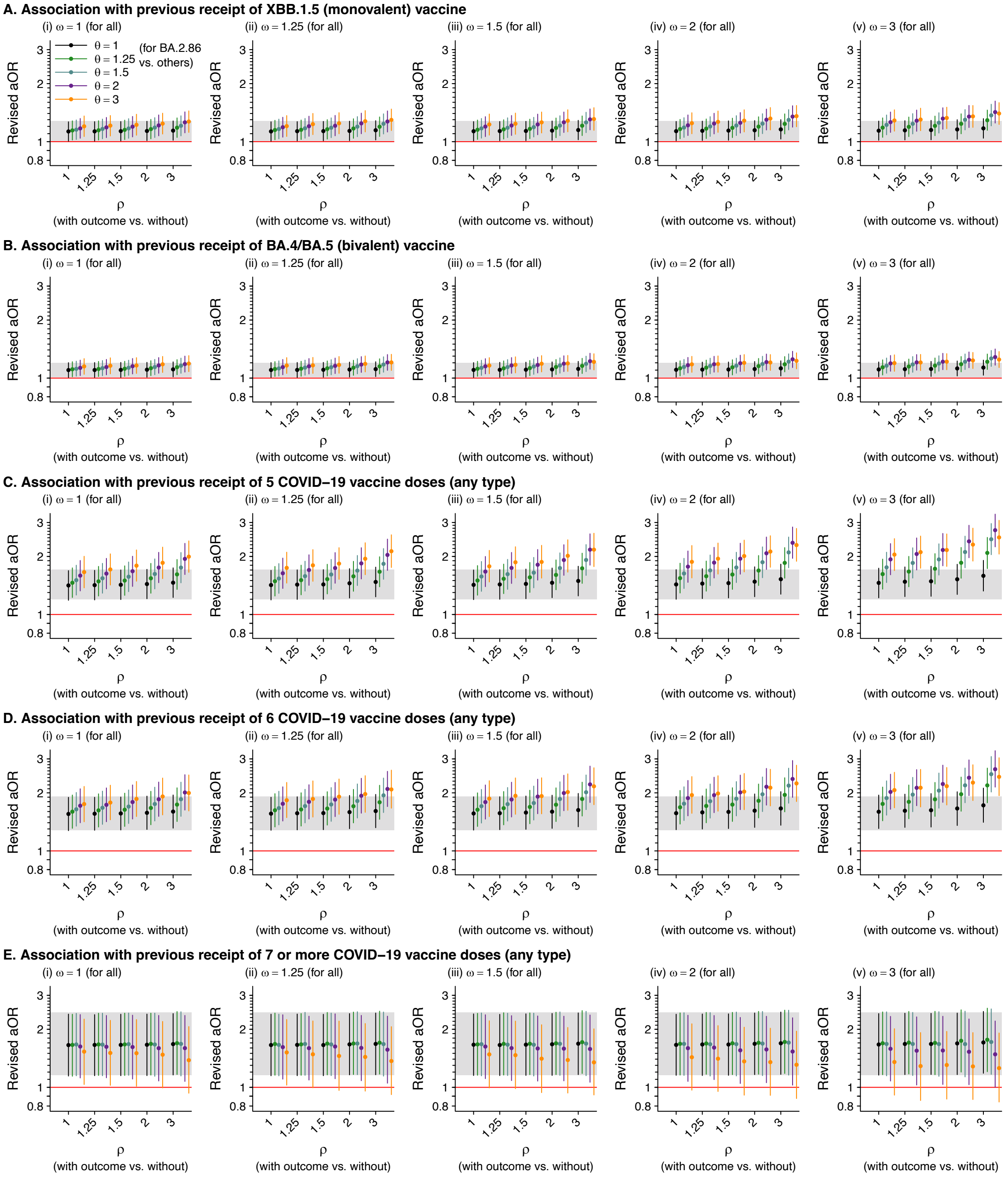

Figure S1: Sensitivity analyses addressing the independent association of BA.2.86 lineage detection with prior vaccination, accounting for additional unobserved infections overall, among cases infected with BA.2.86 lineages, and who did not experience progression (emergency department presentation). We illustrate estimates of the adjusted odds ratio (aOR) of the following vaccine exposures comparing cases infected with BA.2.86 lineages to cases infected with non-BA.2.86 lineages: (**a**) XBB.1.5 (monovalent) vaccine receipt vs. no receipt of this booster; (**b**) BA.4/BA.5 (bivalent) vaccine receipt vs. no receipt of this booster; (**c**) receipt of 5 COVID-19 vaccine doses vs. 0 doses; (**d**) receipt of 6 COVID-19 vaccine doses vs. 0 doses; (**e**) receipt of ≥7 COVID-19 vaccine doses vs. 0 doses. Consistent with the sensitivity analysis addressing progression to severe clinical outcomes (**Figure 4**), we consider multipliers of 1, 1.25, 1.5, 2, and 3 for the ratio of true to observed infections, first non-differentially among all cases (ω), for the relative ratio of true to observed infections comparing cases who evaded progression versus those who experienced to emergency department presentation or higher-acuity levels of illness (ρ), and comparing cases infected with BA.2.86-derived lineages to those infected with non-BA.2.86 lineages (θ). Points and lines illustrate median estimates with accompanying 95% confidence intervals. Grey bands illustrate 95% confidence intervals for estimates from the primary analysis.

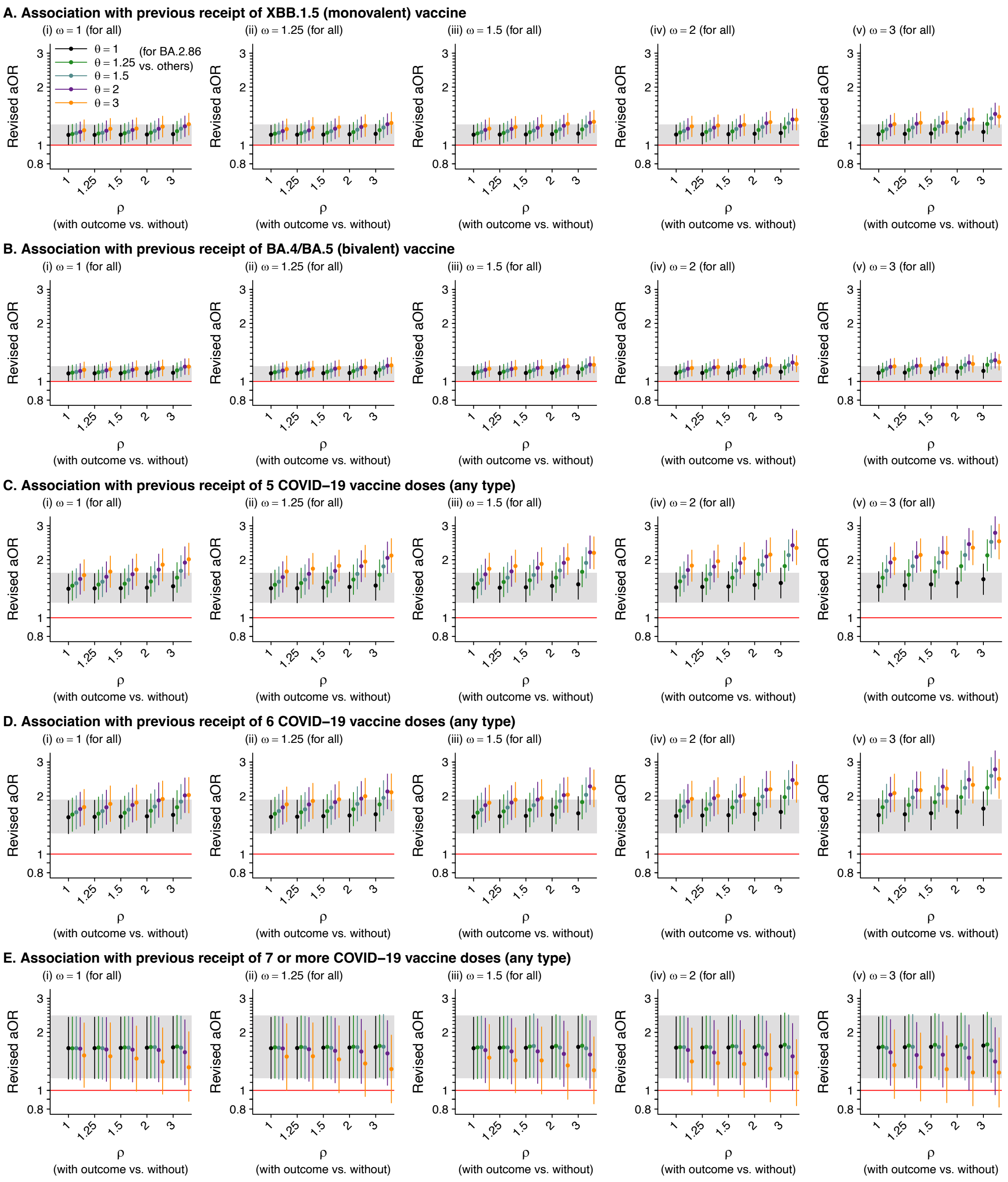

Figure S2: Sensitivity analyses addressing the independent association of BA.2.86 lineage detection with prior vaccination, accounting for additional unobserved infections overall, among cases infected with BA.2.86 lineages, and who did not experience progression (hospital admission). We illustrate estimates of the adjusted odds ratio (aOR) of the following vaccine exposures comparing cases infected with BA.2.86 lineages to cases infected with non-BA.2.86 lineages: (**a**) XBB.1.5 (monovalent) vaccine receipt vs. no receipt of this booster; (**b**) BA.4/BA.5 (bivalent) vaccine receipt vs. no receipt of this booster; (**c**) receipt of 5 COVID-19 vaccine doses vs. 0 doses; (**d**) receipt of 6 COVID-19 vaccine doses vs. 0 doses; (**e**) receipt of ≥7 COVID-19 vaccine doses vs. 0 doses. Consistent with the sensitivity analysis addressing progression to severe clinical outcomes (**Figure 4**), we consider multipliers of 1, 1.25, 1.5, 2, and 3 for the ratio of true to observed infections, first non-differentially among all cases (ω), for the relative ratio of true to observed infections comparing cases who evaded progression versus those who experienced to hospital admission or higher-acuity levels of illness (ρ), and comparing cases infected with BA.2.86-derived lineages to those infected with non-BA.2.86 lineages (θ). Points and lines illustrate median estimates with accompanying 95% confidence intervals. Grey bands illustrate 95% confidence intervals for estimates from the primary analysis.

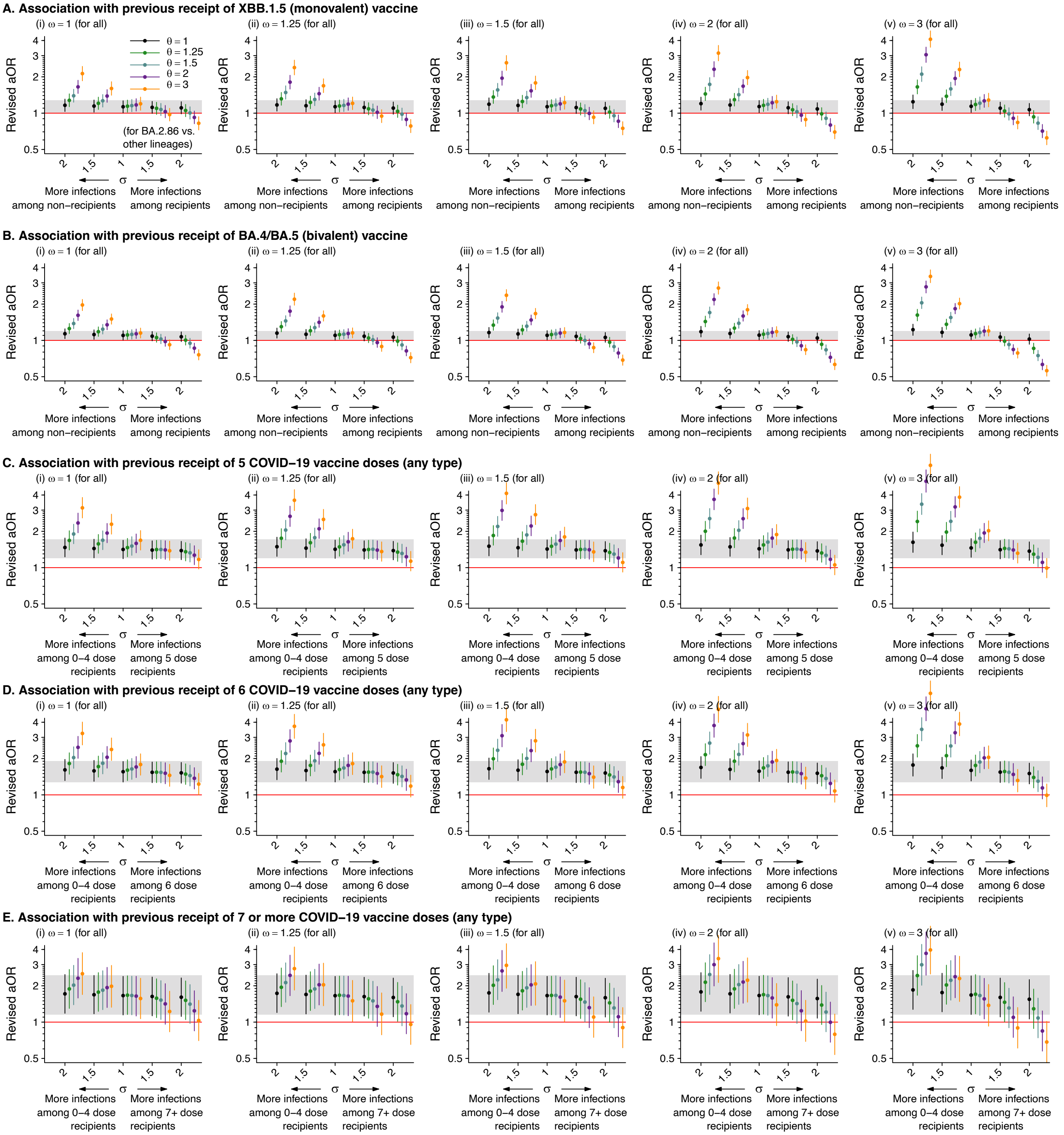

Figure S3: Sensitivity analyses addressing the independent association of BA.2.86 lineage detection with prior vaccination, accounting for additional unobserved infections according to vaccination status. We illustrate estimates of the adjusted odds ratio (aOR) of the following vaccine exposures comparing cases infected with BA.2.86 lineages to cases infected with non-BA.2.86 lineages: (**a**) XBB.1.5 (monovalent) vaccine receipt vs. no receipt of this booster; (**b**) BA.4/BA.5 (bivalent) vaccine receipt vs. no receipt of this booster; (**c**) receipt of 5 COVID-19 vaccine doses vs. 0 doses; (**d**) receipt of 6 COVID-19 vaccine doses vs. 0 doses; (**e**) receipt of ≥7 COVID-19 vaccine doses vs. 0 doses. We consider multipliers of 1, 1.25, 1.5, 2, and 3 for the ratio of true to observed infections, first non-differentially among all cases (ω) and comparing cases infected with BA.2.86-derived lineages to those infected with non-BA.2.86 lineages (θ). Additionally, we consider multipliers of 1, 1.5, and 2 for the ratio of true to observed infections among cases who received or did not receive each vaccine exposure (σ). Points and lines illustrate median estimates with accompanying 95% confidence intervals. Grey bands illustrate 95% confidence intervals for estimates from the primary

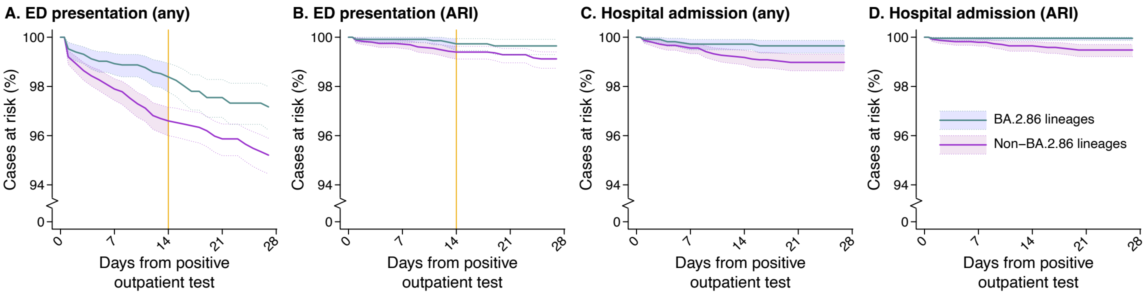

Figure S4: Kaplan-Meier plots for clinical progression analyses comparing cases infected with BA.2.86 lineages to non-BA.2.86 lineages. We present Kaplan-Meier survival curves illustrating the probability over time for cases to evade progression to severe disease necessitating higher-acuity levels of care after an initial positive test in any outpatient setting: (**a**) emergency department (ED), with any diagnosis; (**b**) emergency department, with acute respiratory illness (ARI) diagnosis; (**c**) hospital admission, with any diagnosis; and (**d**) hospital admission, with ARI diagnosis. Analyses included person-time at risk through 14 days after testing for ED presentation outcomes. Lines indicate point (median) estimates, with shaded areas delineating 95% confidence intervals obtained via bootstrap resampling. Additional outcomes listed in **Table 3** (intensive care unit admission, mechanical ventilation, death) are not plotted due to low counts (<10 events within primary analytic sample).

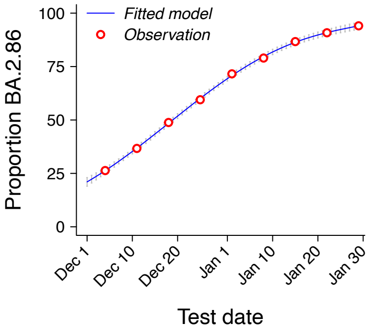

Figure S5: Observed and fitted proportions of cases infected with BA.2.86 lineages. We illustrate weekly proportions of cases found to be infected with BA.2.86 lineages (points, plotted at weekly mid-points), based on sequencing of a random selection of cases across all test settings, against model fitted with a 5th-degree polynomial function. The blue center line corresponds to median estimates, while grey vertical bars delineate 95% confidence intervals in day-specific predictions.
