# Supplementary material for: Immune escape and attenuated severity associated with the SARS-CoV-2 BA.2.86/JN.1 lineage
Source: Nat Commun. 2024 Oct 3;15:8550. doi: 10.1038/s41467-024-52668-w (PMC11450198; doi:10.1038/s41467-024-52668-w)
Supplement: Supplementary file 3 — Reporting Summary [file 41467_2024_52668_MOESM3_ESM.pdf]

nature portfolio

Corresponding author(s): Lewnard, JA

Last updated by author(s): Sep 6, 2024

Reporting Summary

Nature Portfolio wishes to improve the reproducibility of the work that we publish. This form provides structure for consistency and transparency in reporting. For further information on Nature Portfolio policies, see our Editorial Policies and the Editorial Policy Checklist.

Statistics

For all statistical analyses, confirm that the following items are present in the figure legend, table legend, main text, or Methods section.

n/a	Confirmed	
☐	☒	The exact sample size (*n*) for each experimental group/condition, given as a discrete number and unit of measurement
☒	☐	A statement on whether measurements were taken from distinct samples or whether the same sample was measured repeatedly
☐	☒	The statistical test(s) used AND whether they are one- or two-sided<br>*Only common tests should be described solely by name; describe more complex techniques in the Methods section.*
☐	☒	A description of all covariates tested
☒	☐	A description of any assumptions or corrections, such as tests of normality and adjustment for multiple comparisons
☐	☒	A full description of the statistical parameters including central tendency (e.g. means) or other basic estimates (e.g. regression coefficient) AND variation (e.g. standard deviation) or associated estimates of uncertainty (e.g. confidence intervals)
☐	☒	For null hypothesis testing, the test statistic (e.g. *F*, *t*, *r*) with confidence intervals, effect sizes, degrees of freedom and *P* value noted<br>*Give P values as exact values whenever suitable.*
☒	☐	For Bayesian analysis, information on the choice of priors and Markov chain Monte Carlo settings
☒	☐	For hierarchical and complex designs, identification of the appropriate level for tests and full reporting of outcomes
☒	☐	Estimates of effect sizes (e.g. Cohen's *d*, Pearson's *r*), indicating how they were calculated

Our web collection on statistics for biologists contains articles on many of the points above.

Software and code

Policy information about availability of computer code

Data collection	No software was used for data collection in this study.
Data analysis	We conducted analyses using R software (version 4.4.1; R Foundation for Statistical Computing, Vienna, Austria). We used the Amelia package (version 1.8.2) for multiple imputation and fit conditional logistic regression models and Cox proportional hazards models using the survival package (version 3.7-0). Analysis code is available from GitHub (https://github.com/joelewnard/jn1).

For manuscripts utilizing custom algorithms or software that are central to the research but not yet described in published literature, software must be made available to editors and reviewers. We strongly encourage code deposition in a community repository (e.g. GitHub). See the Nature Portfolio guidelines for submitting code & software for further information.

Data

Policy information about availability of data

All manuscripts must include a data availability statement. This statement should provide the following information, where applicable:

- Accession codes, unique identifiers, or web links for publicly available datasets
- A description of any restrictions on data availability
- For clinical datasets or third party data, please ensure that the statement adheres to our policy

The KPSC healthcare system provides managed, integrated care spanning virtual, outpatient, emergency department and inpatient settings to roughly 4.7 million adults residing in southern California, representing roughly 20% of the region’s population. Individuals are enrolled in KPSC plans through employer-sponsored, pre-

paid or government-subsidized coverage schemes. Enrolled members closely resemble the general insured population within Southern California. Electronic health care records capture all in-network care delivery, comprising diagnoses, prescription fills, procedures, laboratory testing, vaccinations, and clinical notes. Records of COVID-19 vaccinations received outside KPSC are imported from the California Immunization Registry. Other care delivered out-of-network is ascertained through insurance claim reimbursements, enabling near-complete ascertainment of members' medical histories. Our study included all individuals who received positive molecular tests for SARS-CoV-2 infection between 1 November, 2023 and 30 January, 2024, who had no clinical record of receiving any positive SARS-CoV-2 test or COVID-19 diagnosis within 90 days before their first test ("index test") within this period, and who were enrolled in KPSC health plans for ≥1 year before their index test (allowing for lapses in membership of up to 45 days). We restricted the primary analytic cohort to individuals tested between 1 December, 2023 and 30 January, 2024 whose index tests were processed using ThermoFisher TaqPath COVID-19 Combo Kit assays, for whom SGTF readout was available. Cases belonging to the primary analytic cohort were predominantly tested in outpatient-serving facilities without in-house laboratory facilities, which relied on regional centers using the ThermoFisher TaqPath COVID-19 Combo Kit for specimen processing. Cases tested in hospital-based emergency departments had specimens processed in hospital laboratories via devices without SGTF readout.

Individual-level testing and clinical outcomes data reported in this study are not publicly shared due to privacy protections for patient electronic health records. Individuals wishing to access disaggregated data, including data reported in this study, should submit requests for access to sara.y.tartof@kp.org. Requests will receive a response within 14 days. De-identified data (including, as applicable, participant data and relevant data dictionaries) will be shared upon approval of analysis proposals with signed data-access agreements in place. Source data to replicate figures are provided with this paper.

Research involving human participants, their data, or biological material

Policy information about studies with human participants or human data. See also policy information about sex, gender (identity/presentation), and sexual orientation and race, ethnicity and racism.

Reporting on sex and gender	Analyses include data on participants' biological sex (male or female).
Reporting on race, ethnicity, or other socially relevant groupings	Analyses include data on participants' race/ethnicity categorized as White non-Hispanic, Black non-Hispanic, Hispanic of any race, Asian, Pacific Islander, or other/mixed/unknown race/ethnicity.
Population characteristics	Characteristics of the population included in analyses are presented in Table 1 (primary analytic cohort) and Table S1 (all outpatients with positive SARS-CoV-2 test results during the study period).
Recruitment	Our retrospective study design used data collected from electronic health records and insurance claim submissions for members of Kaiser Permanente Southern California health plans; no primary recruitment for study participation occurred. Our study included all individuals who received positive molecular tests for SARS-CoV-2 infection between 1 November, 2023 and 30 January, 2024, who had no clinical record of receiving any positive SARS-CoV-2 test or COVID-19 diagnosis within 90 days before their first test ("index test") within this period, and who were enrolled in KPSC health plans for ≥1 year before their index test (allowing for lapses in membership of up to 45 days). We restricted the primary analytic cohort to individuals tested between 1 December, 2023 and 30 January, 2024 whose index tests were processed using ThermoFisher TaqPath COVID-19 Combo Kit assays, for whom SGTF readout was available. Cases belonging to the primary analytic cohort were predominantly tested in outpatient-serving facilities without in-house laboratory facilities, which relied on regional centers using the ThermoFisher TaqPath COVID-19 Combo Kit for specimen processing. Cases tested in hospital-based emergency departments had specimens processed in hospital laboratories via devices without SGTF readout.
Ethics oversight	This study was reviewed by the Institutional Review Board of Kaiser Permanente Southern California and the US Centers for Disease Control and Prevention (CDC) and was conducted consistent with applicable federal law and CDC policy (45 CFR part 46, 21 CFR part 56; 42 USC Sect. 241 (d); 5 USC Sect. 552a; 44 USC Sect. 3501 et seq.).

Note that full information on the approval of the study protocol must also be provided in the manuscript.

Field-specific reporting

Please select the one below that is the best fit for your research. If you are not sure, read the appropriate sections before making your selection.

☒ Life sciences ☐ Behavioural & social sciences ☐ Ecological, evolutionary & environmental sciences

For a reference copy of the document with all sections, see nature.com/documents/nr-reporting-summary-flat.pdf

Life sciences study design

All studies must disclose on these points even when the disclosure is negative.

Sample size	The study used passively collected, retrospective data from all participants meeting eligibility criteria. Because the study did not involve prospective recruitment, the investigators did not pre-determine a targeted sample size a priori. Numbers of research subjects involved in the study were determined by the number of patients diagnosed with SARS-CoV-2 infection during the study period (1 November, 2023 to 30 January, 2024) who met criteria listed above; this totaled 46,067 individuals of whom 7,647 had tests processed via assays that provided S-gene target failure readout for lineage calling. This sample size was adequate to provide >80% power for estimation of odds ratios of 1.10 or more at two-sided p=0.05 for the key exposures of interest (prior XBB.1.5 monovalent vaccination and BA.4/BA.5 bivalent vaccination, comparing BA.2.86/JN.1 infections to non-BA.2.86/JN.1 infections).
Data exclusions	Our study was restricted to patients testing positive in outpatient settings in order to provide an opportunity for longitudinal follow-up over which progression could be monitored for hospital admission (indicative of an escalation in care). Patients whose initial positive test occurred in an inpatient setting were thus ineligible for inclusion. In addition, we excluded patients without ≥1 year of prior membership in KPSC health

plans in order to ensure comprehensive capture of relevant medical history including comorbid conditions and prior COVID-19 vaccination. Last, our primary analytic cohort was restricted to individuals whose tests were processed using the TaqPath COVID-19 Combo Kit in order to obtain individual-level determinations of S gene target failure and thus call out specific infecting lineages.

Replication	While our design did not include technical replication, we used multiple analysis approaches to confirm the primary findings. Specifically, we compared results of our primary analyses (comparing outcomes and vaccination/prior infection status among cases infected with BA.2.86 lineages and XBB lineages) to results of analyses comparing a broader sample of cases, for whom individual-level lineage determinations were not possible, who were infected during eras when BA.2.86 lineages and XBB lineages were predominant. These analyses yielded conceptually similar findings. Cases infected with BA.2.86 lineages or during BA.2.86-dominant periods experienced lower risk of adverse clinical outcomes, had received greater numbers of prior COVID-19 vaccine doses, and had experienced greater number of prior documented SARS-CoV-2 infections.
Randomization	Our study was observational in nature, and randomization was not possible. Regression models for all analyses controlled for age, sex, race/ethnicity, body mass index, history of cigarette smoking, prior-year healthcare utilization across all settings, Charlson comorbidity index, and median household income within cases' census tract. For analyses within the primary analytic cohort (for whom individual-level lineage calling was possible), models further matched on cases' week of testing.
Blinding	Participants and clinicians did not have access to information on infecting lineages. Thus, this exposure was blinded and could not inform care management. For analyses reported in this manuscript, investigators were not blinded to any participant characteristics.

Reporting for specific materials, systems and methods

We require information from authors about some types of materials, experimental systems and methods used in many studies. Here, indicate whether each material, system or method listed is relevant to your study. If you are not sure if a list item applies to your research, read the appropriate section before selecting a response.

Materials & experimental systems

n/a	Involved in the study
☒	☐ Antibodies
☒	☐ Eukaryotic cell lines
☒	☐ Palaeontology and archaeology
☒	☐ Animals and other organisms
☐	☒ Clinical data
☒	☐ Dual use research of concern
☒	☐ Plants

Methods

n/a	Involved in the study
☒	☐ ChIP-seq
☒	☐ Flow cytometry
☒	☐ MRI-based neuroimaging

Clinical data

Policy information about clinical studies

All manuscripts should comply with the ICMJE guidelines for publication of clinical research and a completed CONSORT checklist must be included with all submissions.

Clinical trial registration	N/A (not a clinical trial)
Study protocol	N/A (not a clinical trial)
Data collection	Our study included all individuals who received positive molecular tests for SARS-CoV-2 infection between 1 November, 2023 and 30 January, 2024.
Outcomes	We followed cases for the following outcomes within the specified time range from the index test: any emergency department presentation within 14 days; ARI-associated emergency department presentation within 14 days; any hospital admission within 28 days; ARI-associated hospital admission within 28 days; ICU admission within 60 days; initiation of mechanical ventilation within 60 days; and death within 60 days. Due to the low frequency of severe outcomes, we defined a composite outcome of ICU admission, initiation of mechanical ventilation, or death within 60 days.

Plants

Seed stocks	N/A
Novel plant genotypes	N/A
Authentication	N/A
